# Investigating the Water Relations in Aqueous Extract Powders of Mango (*Mangifera indica*) Peel and Seed Waste for Their Use in Food Matrices as a Value-Added By-Product

**DOI:** 10.3390/foods12183497

**Published:** 2023-09-20

**Authors:** Ronald Marsiglia-Fuentes, Amparo Chiralt, Luis A. García-Zapateiro

**Affiliations:** 1 Research Group on Complex Fluid Engineering and Food Rheology (IFCRA), Faculty of Engineering, Department of Food Engineering, University of Cartagena, Consulate Avenue, St. 30 No. 48-152, Cartagena 130015, Colombia; rmarsigliaf@unicartagena.edu.co; 2 Food Technology Department, Food Engineering Institute for Development, Universitat Politècnica de València, Camino de Vera s/n, 46022 Valencia, Spain; dchiralt@tal.upv.es

**Keywords:** mango (*Mangifera indica*), waste products, aqueous extracts, glass transition, functionality

## Abstract

This study investigated the potential uses of discarded mango peel and seed parts by analyzing their water sorption behavior, hydration kinetics, and stability when converted into extract powders at pH 3 and 10. The results revealed that peel extracts had a higher water adsorption capacity compared with seed extracts due to differences in their composition. Peel extracts were primarily composed of carbohydrates (approximately 75%) with a low protein content, while seed extracts contained fewer carbohydrates (less than 30%) but higher levels of proteins (more than 30%) and lipids. The critical water content for maintaining the glassy state of peel extract powders during storage was found to be 0.025 and 0.032 g of water/g for extracts obtained at pH 3 and 10, respectively. In contrast, the T_g_ values of seed extracts remained relatively unchanged across different water content levels, suggesting that proteins and lipids inhibited the water’s plasticizing effect in the solid matrix. These findings indicate that both mango waste fractions exhibit distinct hygroscopic behaviors, necessitating different approaches to processing and utilization. These extracts hold potential applications for various food products such as beverages, gels, sauces, or emulsions, contributing to the reduction in waste and the creation of value-added products from mango residues.

## 1. Introduction

The sustainable development of food products has become a priority issue for government agencies due to its positive impacts on social, environmental, and economic factors [1,2]. As reported by Socas-Rodríguez et al. (2021) [3] and FAO (2019) [4], the post-harvest food loss rates are around 30%. Roots, tubers, and oilseeds show a loss of 25–30%, while fruits and vegetables experience a 22% loss. Meat and animal products have a loss rate of 12%, and cereals and legumes have a lower loss percentage of 9%; fruit waste presents an opportunity to extract economically valuable substances, including organic acids, proteins, essential oils, enzymes, bioactive compounds, aromatic substances, cellulose, pectin, and polysaccharides. These substances contribute to the economic value derived from fruit waste [5,6,7,8]. The proper utilization of fruit waste has the potential to significantly reduce challenges of overproduction, particularly in countries where these fruits are widely grown. By effectively managing and leveraging fruit waste, through methods such as recycling, composting, or extracting valuable substances, one can mitigate the environmental and economic impacts of surplus fruits. This approach promotes resource efficiency, minimizes waste, and promotes a more sustainable agricultural system in regions heavily involved in fruit production [9] by using surplus stocks as a resource to obtain valuable compounds for the formulation of new products and promoting an appropriate natural cycle of materials [10].

Mango (*Mangifera indica* L.) is a member of the Anacardiaceae family and is found naturally in tropical and subtropical regions around the world [11]. In recent years, mango cultivation has expanded to several European regions located near the Mediterranean Sea due to their subtropical climate, which is favorable to growing and harvesting this fruit. Additionally, mangoes have gained global popularity, driven not only by their delicious aroma and taste but also by the presence of bioactive compounds that offer significant nutritional value and nutraceutical properties. These attributes make mangoes valuable for use in both food and industrial products, creating a growing demand worldwide [12,13,14]. More than 55 million tons are produced annually, and mango is considered the second most important tropical fruit with a high global demand [15]. However, mango is highly perishable due to its high moisture content, resulting in significant post-harvest losses [16]. Despite the economic benefits, mango processing is perceived as a residue industry with a waste percentage of approximately 30% [17], mainly from the peel and seeds [18]. These residues contain valuable fractions that can be extracted by aqueous extraction systems [19]. The aqueous method for extraction is excellent for the extraction of proteins, carbohydrates, enzymes, and other labile biomolecules from crude cell extracts or mixed biological materials [20]. Some interesting examples of this developing field include the removal of dyes from textile effluents, metal ions and organic compounds from the environment, and aromatic compounds from crude oil [21,22].

The characterization of the product from the perspective of thermal analysis is essential for estimating stability in terms of changes in temperature and kinetic parameters when referring to thermogravimetric analysis (TGA) [23]. In several studies, this has been implemented as the main requirement for identifying the thermal stability of materials and determining the degradation peaks (the temperature at which degradation is maximal) [24,25,26,27], and used as a useful tool for estimating drying temperatures [26]. Another thermal analysis technique is differential scanning calorimetry (DSC), a method in which the difference in heat flux rate between the reference material and the product being evaluated is estimated as a temperature element, while the substance and the reference are subjected to a manageable temperature program ranging from subzero to high temperatures [28]. The DSC technique allows for the determination of the phase transition temperature, the polymorphic transformation, and the glass transition temperature [29]. To determine the stability of materials, the sorption isotherm is also used, which graphically represents the moisture content of a substance in relation to its water activity under isothermal conditions (hygroscopic equilibrium) with the environment when the weight of the sample does not vary [30]; these provide information about the degree of crystallinity and the motion of the water, which differ greatly in crystalline and amorphous systems. In the amorphous system, the molecules are randomly arranged and relatively free to interact with water. The absorption temperature of water is a suitable method for analyzing the interaction of water with a substrate. In general, it can be divided into three ranges depending on a_w_: strongly bound water corresponding to a_w_ ≤ 0.3, moderately bound water (a_w_ = 0.3–0.7), and weakly bound water corresponding to a_w_ 0.7–0.8 and higher [31]. The removal of water during drying promotes the formation of an amorphous matrix in which water-compatible soluble and insoluble biomaterials are molecularly disordered [32]. Non-equilibrium thermodynamic states demonstrate time-dependent changes that gradually approach equilibrium. Amorphous materials can exist in either a glassy or rubbery state, with the glass transition temperature (T_g_) denoting the transition between these states [33,34]. The aim of this work was to evaluate the correlations among water activity, moisture content, and thermal stability of extract powders derived from mango (*Mangifera indica*) peel and seed.

## 2. Materials and Methods

### 2.1. Material

Mango (*Mangifera indica*) of the Tommy variety was bought at the local market in Cartagena de Indias, Colombia, with organoleptic maturity. The fruits were conditioned and washed with sodium hypochlorite (0.1%) for 10 min at a temperature of 65 °C followed by manual separation of pulp, seed, and peel using stainless steel knives. The seeds and peel were rinsed to remove any remaining adherent pulp. The raw material was then frozen and lyophilized using Labconco Freezone 1.5 L benchtop equipment for 48 h at −50 °C and 0.02 kPa and subsequently ground in an IKA MF 10.2 mill connected to a sieve.

### 2.2. Chemical Reagents

Analytical-grade chemicals Ethanol, Glacial Acetic Acid and Hexane (99.5%), from Panreac (Barcelona, Spain), sodium hydroxide (NaOH, pellet for analysis), buffer solutions (boric acid/potassium chloride/sodium hydroxide), sodium azide, and phenolphthalein were purchased from Sigma-Aldrich (St. Louis, MO, USA).

### 2.3. Extraction Process

The powder extracts were produced according to the conditions established in a previous study [35], based on the procedures described by López-Barraza et al. [36] and Quintana et al. [37], with some modifications. The mango waste powder (peel and seeds) was dispersed in distilled water, previously adjusted at pH 3 and 10, with 1 N acetic acid and 0.1 N sodium hydroxide, respectively, in a 1:8 ratio at a constant temperature of 80 °C. The dispersion was stirred for 4 h and filtered to remove the insoluble material. The obtained solution was mixed with ethanol (1:1) at −1 to −40 °C with constant stirring for 3 h to promote precipitation. Finally, the precipitated products were obtained by centrifugation (4000 rpm at room temperature) and freeze-dried for 72 h at −50 °C and 0.02 kPa using freeze-drying equipment (Labconco Freezone 1.5 L Benchtop). Four extract powders were obtained this way: peel and seed at ph 3 (E3); (S3) and 10 (E10); (S10), respectively.

### 2.4. Sample Composition

Moisture, lipids, ash, and protein content were determined using standard AOAC methods 926.08, 972.28, 935.42, and 926.123, respectively, described in the standard methods of the Association of Official Analytical Chemists [38]. Additionally, FTIR spectra of each sample were obtained (FT/IR-4100 TYPE A JASCO, Barcelona, Spain), ranging from 500 to 4000 cm^−1^ infrared region with a resolution of 4 cm^−1^ via 32 average scans.

### 2.5. Water Sorption Isotherms

Samples of powders extracted from the peel and seed (at pH 3 and 10) were accurately weighed, and the equilibrium moisture content was determined using a vacuum oven at 60 °C and 6.6 kPa for 2 days; then, different samples were placed in desiccators at 25 °C at different relative humidities and analyzed via the static gravimetric method [39], containing oversaturated solutions between 0.11 and 0.75 using LiCl (a_w_—0.11), MgCl_2_ (a_w_—0.33), K_2_CO_3_ (a_w_—0.46), Mg (NO3)_2_ (a_w_—0.53), and NaCl (a_w_—0.75) [33]. The samples were periodically weighed with a calibrated analytical balance until each sample reached a constant weight when the equilibrium moisture content was weighed with its respective a_w_ value. Finally, the experimental data were fitted to the Guggenheim Anderson deBoer (GAB) model (Equation (1)), and widely used in the full a_w_ range.

(1) $$W_{e}=\frac{w_{o}\cdot C\cdot K\cdot a_{w}}{\left(1-K\cdot a_{w})\cdot (1+(C-1)\cdot K\cdot a_{w}\right)}$$

In Equation (1), W_e_ correspond to the equilibrium moisture content (dry basis), W_o_ corresponds to monolayer moisture and C and K are the equation parameters, both dependent on temperature and related to the sorption water energy.

The Peleg model (Equation (2)) was used to describe the hydration kinetics of powder extracts [40], where M_t_ is the moisture content dry basis (g water/g anhydra sample) after time ‘t’, M_0_ is the initial water content of the sample (g water/g anhydra sample), t is the exposure time (h), K_1_ is inversely related to the initial mass transfer rate [day (g/g)^−1^], and K_2_ [(g/g)^−1^] is inversely related to the asymptotic value of the function and therefore, to the equilibrium moisture content.

(2) $$M_{t}=M_{0}+\frac{t}{K_{1}+K_{2}\cdot t}$$

### 2.6. Calorimetric Analyses

#### 2.6.1. Thermogravimetric (TGA) Analysis

To determine the thermal behavior of mango peel and seed powder extracts at pH 3 and 10, a TGA analyzer (TGA1 Stare System analyzer, Mettler-Toledo, Greifensee, Switzerland) was used under nitrogen flow (10 mL/min). The samples were weighed (approximately 3–5 mg) in an alumina pan and heated from 25 to 700 °C at 10 °C/min. Before analyses, the samples were conditioned in a desiccator with P_2_O_5_ at room temperature for 2 weeks. Thermogravimetric curves (TGA) and their derivative curves (DTGA) were analyzed using STARe evaluation software version 11 for Windows (Mettler-Toledo, Greifensee, Switzerland) to obtain the initial temperature (T-onset), the maximum degradation rate temperature (T-peak), and the percentage mass loss in each thermal event [41]. Each sample was analyzed in duplicate.

#### 2.6.2. Differential Scanning Calorimetry (DSC) Analysis

Differential scanning calorimetry (DSC) was performed using a DSC 1 stareSystem, Mettler Toledo, Schwarzenbach, Switzerland, with a constant heating or cooling rate (10 °C/min). Samples (8–10 mg) equilibrated at different relative humidities were placed in aluminum pans, and an empty aluminum pan was used as a reference. The temperature scanning profile used to analyze the phase transitions was as follows: first, heating from −40 °C to 170 °C, holding this temperature for 5 min, subsequent cooling to −40 °C, holding this temperature for 5 min; and second, heating to 170 °C. Finally, the temperature of T_g_ was determined to be the midpoint temperature of the glass transition. Each sample was analyzed in duplicate. Gordon and Taylor model equation (Equation (3)) was fitted to the experimental data T_g_ vs. moisture content (x_w_) [33,41,42].

(3) $$T_{g}=\frac{\left(1-x_{w}\right)\cdot T_{g\left(as\right)}+K\cdot x_{w}\cdot T_{gw}}{\left(1-x_{w}\right)\cdot +K\cdot x_{w}}$$

#### Statistical Analysis

Data were analyzed via ANOVA (unidirectional) using SPSS software (version 17.2 for Windows) to determine statistically significant differences (*p* < 0.05) between samples.

## 3. Results and Discussion

### 3.1. Sample Composition

The samples obtained have the characteristic in common that they are mostly waste materials, which can be used as an alternative in the industrial sector. The chemical composition depends on the sample and extraction conditions, and Table 1 shows the composition of the seeds and mango peel extracts obtained at different pH values. The moisture content ranged from 11 to 13%, while the ash content was ±9%, with little difference between samples on a dry basis. On the contrary, the lipid content was much higher in the seed samples (18%) than in the peel samples (3%). Similarly, the seed extracts were richer in proteins (31–32%) than the peel extracts (0.35%). Carbohydrates (obtained as a difference) were present mainly in peel extracts (73–76%), while their content was much lower in seed extracts (28%). The effect of extraction pH on the composition of the peel and seed extracts was not significant in most cases, as observed in previous studies [35]. Therefore, the seed extracts (S3 and S10) were richer in lipids and proteins, while the peel extracts (E3 and E10) contained mainly carbohydrates. This compositional characteristic may lead to the formulation of new food products with functional properties of great importance [36,43]. Bertuzzi et al. [44] and Chaudhari et al. [45] extracted and characterized hydrocolloids from plant raw materials such as Astragalus gum exudates and *Limonia acidissima* L. gum exudates, obtaining similar results in terms of carbohydrate content in powdered extracts.

**Figure 1:**
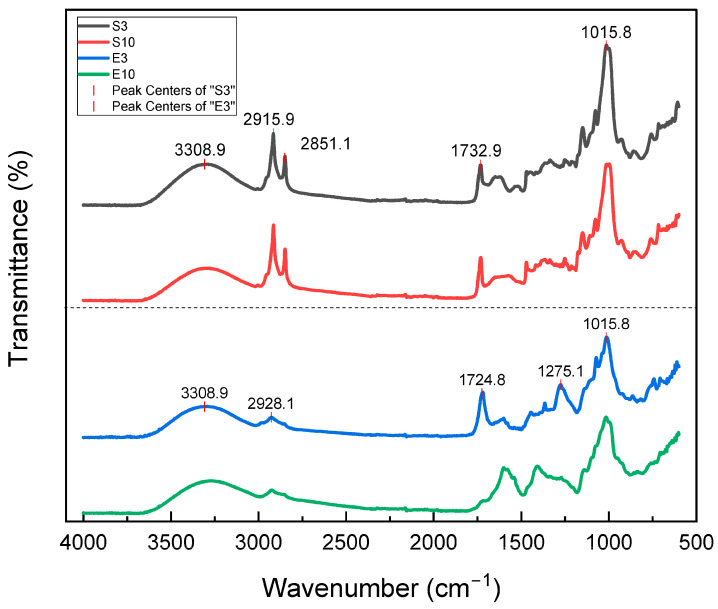
FTIR spectra of the peel and seed extract samples.

The FTIR spectra of freeze-dried extract powders from mango peel and seed are shown in Figure 1. All samples exhibited bands at 3308–3360 cm^−1^, which is attributed to O–H stretching in intramolecular and intermolecular hydrogen bonds. The bands between 2800 and 3000 cm^2^ were attributed to the vibrations of symmetric stretching of the C-H, which were more intense in seeds (S3 and S10) obtained according to the fat content and the presence of more fatty acids [46]. In all samples, the band at ~1730 cm^−1^ is associated with the stretching vibration of the C-O bond due to non-ionic carboxyl groups or their ester acids (–COOH, –COOCH_3_) [47]; The typical bands at ~1611 cm^−1^ and 1415 cm^−1^, attributed to the symmetric and asymmetric stretching vibrations of –COO of uronic acids, respectively, were also found [48]. Characteristic peaks of the polysaccharide molecules [49] in the 1015 cm^−1^ band are assigned to the C-O and C-C stretching vibrations and C–O, C–O–C glycosidic, and C–O–H bonds [50,51].

The relative Intensity of the peaks between 1275 and 1375 cm^−1^ may be assigned to the symmetric pectin -COO stretching vibrations in the peel, while the symmetric and asymmetric stretching vibrations of ionic carboxylic groups (–COO–), respectively, appeared in E10 at 1400 and 1620 cm^−1^ [52]. This, in addition to the -COO- groups of the molecular chain, improves the ability to retain water and interact to form more stable gels, which occur mostly when there are amorphous regions [53].

### 3.2. Water Sorption Isotherms

**Figure 2:**
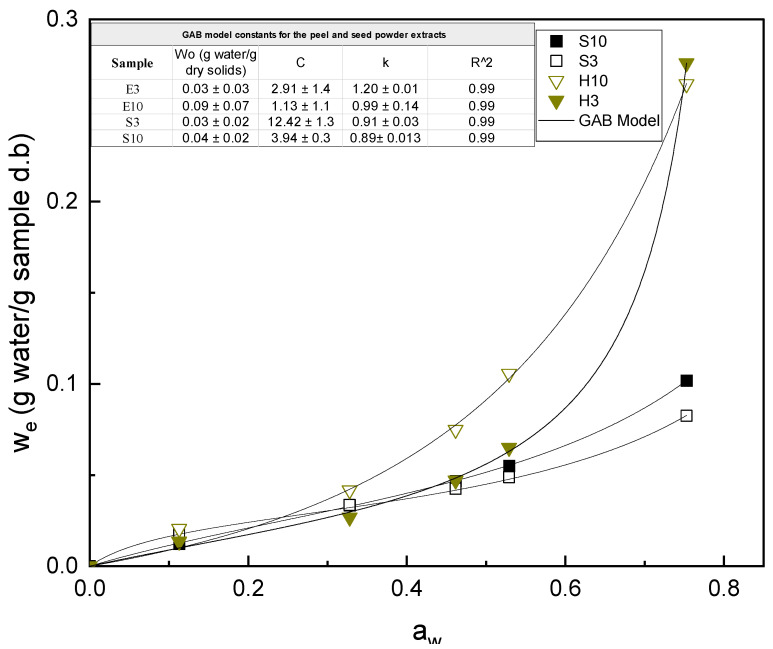
Water sorption isotherms of the peel and seed extracts at different pHs. Experimental points and GAB-fitted model. The embedded table shows the GAB parameters for each sample.

The sorption isotherms of the sample powders extracted at pH 3 and 10 from mango peel and seed are shown in Figure 2, where the equilibrium moisture content was plotted as a function of the conditioning water activity. The obtained curves exhibited the typical behavior of type II for the peel and type III for the seed of a sigmoidal sorption curve exhibited by polysaccharides [54,55]. In this type of adsorption behavior, water molecules are adsorbed in multiple layers, consistent with data available in the literature [54,56]. Remarkable differences can be observed between the peel and seed extracts, the former showing an equilibrium moisture content of w_e_ > 0.24. This can be attributed to the higher content of carbohydrates with a more hydrophilic nature and water sorption capacity [57]. On the contrary, the seed extract mainly contains proteins and fats, more hydrophobic in nature, with a lower water affinity and sorption capacity, with w_e_ < 0.1, which can even create barriers that inhibit the hydration of the extract [58,59]. The embedded table in Figure 2 shows the parameters of the GAB model fitted to the experimental data (R^2^ > 0.996). The constants *C* and *K* related to the sorption enthalpy are in the range of those previously obtained for freeze-dried carbohydrate blend powders [60].

**Figure 3:**
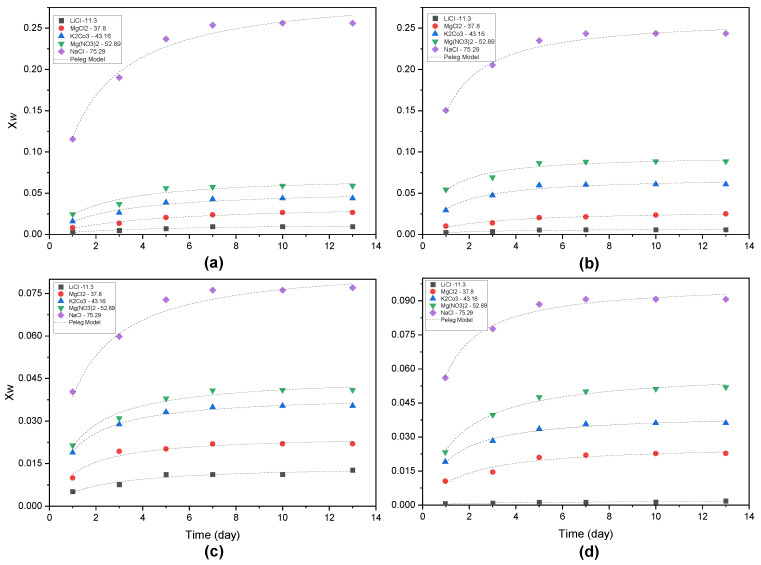
Water uptake kinetics (water mass fraction vs. time) at 25 °C of the different extract powders from the mango seed and peel samples: (**a**) E3, (**b**) E10, (**c**) S3, (**d**) S10.

The experimental data on the hydration kinetics of the different mango extract samples in terms of the fraction of water mass of the samples as a function of exposure time at different relative humidities are shown in Figure 3. Based on the results obtained, samples E3 and E10 slowly hydrate in the first three days in all chambers with different salts, and then the hydration rate increases after day three. However, as the a_w_ increases to 75.29 (Nacl chamber), the X_w_ also increases significantly until reaching values of 0.25, suggesting that the material has significantly increased its capacity to absorb water. On the other hand, samples S3 and S10 present the same nonlinear hydration kinetics behavior, but their capacity to absorb water is reduced, having maximum X_w_ values of 0.075 for S3 and 0.09 for S10. In general, equilibrium moisture for all samples in the different salt chambers is reached within seven (7) days.

The Peleg model [59] was fitted to each series of experiments, and the parameters are shown in Table 2. As the relative humidity of the environment increased, the water sorption capacity of the samples increased, as indicated by the sorption isotherms, which provides information on the strength of interaction between water and the sample matrix [58]. The values of the coefficient of determination (R^2^) ranged from 0.87 to 0.99 with *p* < 0.05, indicating that this model can be used to predict water uptake as a function of time in RH. K_2_ is inversely related to the maximum water uptake capacity [55,61] of the different samples extracted from the mango peels and seeds. K_1_ is inversely related to the initial water transfer rate. As expected, the initial hydration rate increased when RH increased, in accordance with the higher driving force of hydration [62,63], but the seed samples had lower hydration rates (1/K_1_), with maximum values for the NaCl chamber at 75.28% RH; the hydration capacities of E3 and E10 were determined to be 0.195 and 0.166, respectively, while S3 and S4 had hydration capacities of 0.061. These values confirm that the peel extracts have an increased capacity to absorb water more rapidly. Furthermore, this water absorption capacity (1/K_2_) is directly proportional to the calculated maximum values of 0.296 and 0.263 for E3 and E10, respectively, while S3 and S10 showed values of 0.086 and 0.098. It should be noted that there is no significant tendency for these values to increase with changes in RH. Similarly, the equilibrium moisture content at each RH was higher in the peel extract powders than in those from the seed, as already explained in the section on water sorption. However, the differences were more pronounced when RH increased, at which point the peel samples had much higher hydration rates and asymptotic values. Therefore, the seed extracts exhibited a less hygroscopic nature, with lower values of the hydration rate and equilibrium moisture value.

### 3.3. Phase Transitions

**Figure 4:**
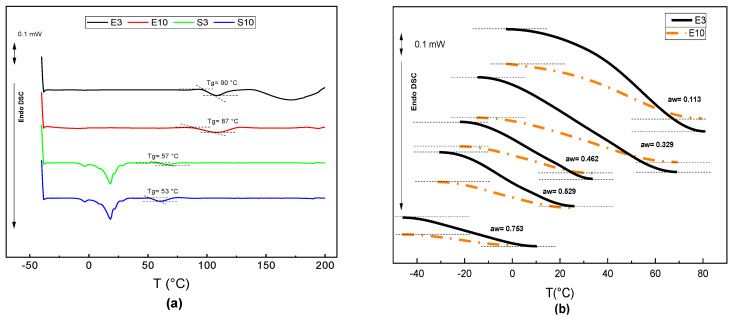
Typical thermograms (second heating) of the different freeze-dried samples (**a**) and glass transitions of the peel samples (E3 and E10) equilibrated at different a_w_ values (**b**).

The DSC thermograms obtained for the anhydrous powder extracts of peel (E3; E10) and seed (S3; S10) in the second heating step are shown in Figure 4a. Typical curves of the second-order transition (glass transition) were observed for every sample. The midpoint T_g_ values were between 87 and 90 °C in the peel extract samples and between 53 and 57 °C for the seed extract samples. Compared with the values of other polysaccharides such as gellan gum [64], with a T_g_ of 77 °C, the values of the peel extract are similar, but those obtained for the seed extracts are lower.

The expected decrease in the T_g_ values of the peel extracts with increasing moisture content, when conditioned to a higher RH, can be seen in Figure 4b. The decrease in T_g_ values with increasing sample water content is related to the plasticization of powders containing polysaccharides in the water and to the subsequent increase in molecular mobility in the solid sample [65]. The extraction pH of the samples did not have a significant effect on the phase transitions, which is in agreement with the small changes in composition promoted by the extraction pH. The minimum value of T_g_ (−18 °C) was reached for the E3 and E10 samples equilibrated at a_w_ = 0.763. In contrast, the seed extracts (S3 and S10) did not show significant changes in glass transition temperature when their water content or a_w_ value increased. This behavior could be related to the predominant presence of lipids and proteins, which are less sensitive to water and can even act as a barrier to limit the plasticization effect of water [57,66,67]. The melting endotherm associated with the melting of the lipids was also observed in the heating step of the seed extract samples (Figure 4a). The lipid melting started at 12.3 °C, with a maximum peak at 18.5 °C, and a final melting at 25.36 °C. The results are similar to those reported by Lieb et al. [68] for the melting properties of fat obtained from mango seeds, which were between 9.6 and 18.5 °C. The melting enthalpy of the mass fraction of fat in the powder extracts was 260.94 j/g for S3 and 304.96 j/g for S10. The extraction conditions, in terms of pH, did not produce significant variations.

**Figure 5:**
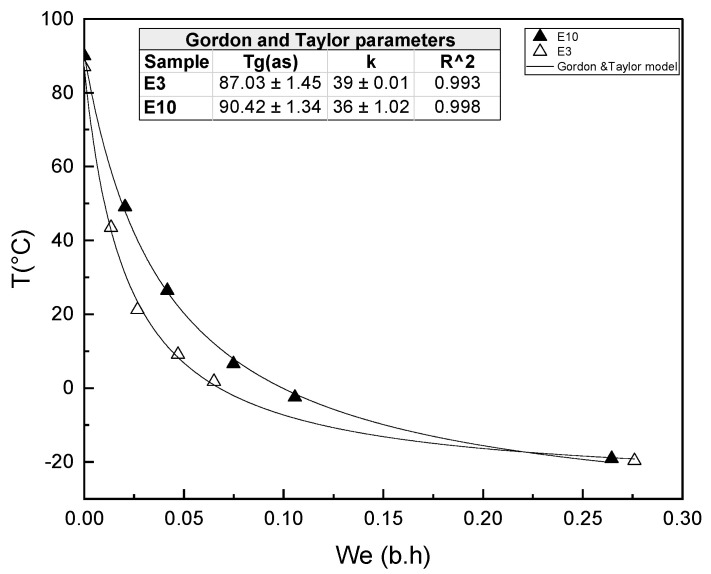
Values of the T_g_ versus equilibrium water content (g water/g sample w.b) for the E3 and E10 samples.

The relationship between T_g_ and the equilibrium moisture content of the powder extracts obtained from the peel at pH 3 and 10 is plotted in Figure 5, where the plasticizing effect of water can be observed for both samples. This behavior was fitted by the Gordon and Taylor equation (R^2^ > 0.99) and the parameters obtained (empirical constant K and T_g_ of anhydrous solids (T_g_s)) are shown in the embedded table. The K value was similar in samples 39 (E3) and 36 (E10), while the glass transition temperatures of the anhydrous extracts E10 (90.4 °C) and E3 (87.0 °C) coincide with the experimental values. The extraction pH affected the water plasticization effect, with the sample obtained at pH 3 being slightly more sensitive with higher values of the water plasticization rate (K). The T_g_ values of the anhydrous mango peel extract were slightly higher than those of previously reported values for the powder of mango fruits with maltodextrin [69], whose main components are sucrose (0.064 g/g dry solid), fructose (0.026 g/g dry solid), and glucose (0.047 g/g dry solid), with T_g_ values (as) of 62 °C, 5 °C, and 31 °C, respectively. This suggests that the mango peel extracts contained carbohydrates of higher molecular weight.

**Figure 6:**
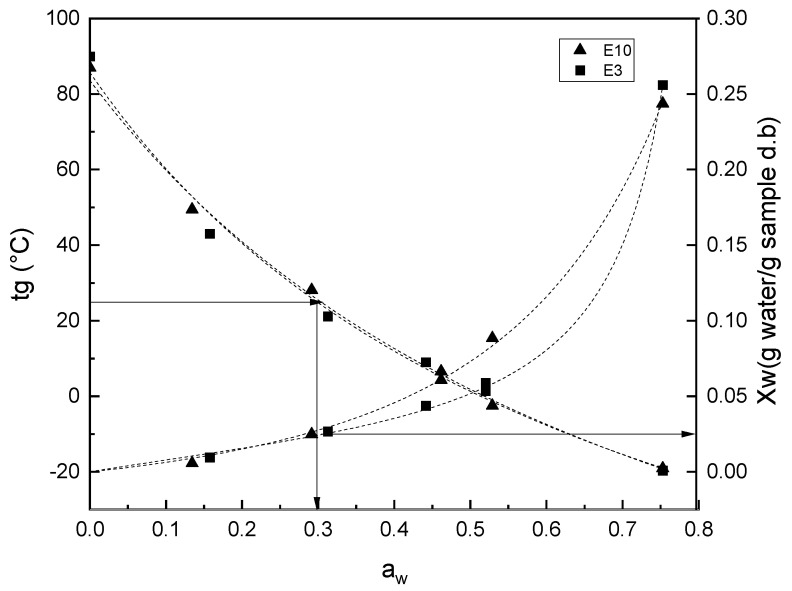
Glass transition water activity and water content relationships of mango peel extract powders obtained at extraction pH of 3 and 10.

Critical water content (CWC) and critical water activity values (CWA) related to glass transition values for the samples E3 and E10 were obtained from the combined plot of T_g_ − x_w_ − a_w_, with the corresponding models fitted with models fitted with GAB and Gordon and Taylor fitted models (Figure 6). This graph corresponds to a state diagram showing the relationship between the water content in the extract powders and its physical state as a function of temperature [33]. This diagram allows for predicting the critical values of the water content and a_w_ at which the change of state (glassy to rubbery) would occur at the corresponding temperature of the sorption data [34,70]. Therefore, at 25 °C (near room temperature), the CWA for the glass transition of the peel extract, considering the midpoint, was 0.025 and 0.032 for E3 and E10, respectively. Therefore, the maximum relative humidity of the atmosphere that would ensure the glassy state of the powders throughout the storage period is 2.5% and 3.2%, respectively. The corresponding CWC was 5.1 g water/100 g sample in E3 and 4.7 g water/100 g sample in E10. The low critical values obtained for extract powders justify the use of high molecular weight solutes, such as polysaccharides with a large chain, e.g., arabic, xanthan, or guar gum; in order to increase the T_g_ of the product and promote stability during handling and storage. Therefore, the combined analysis of the T_g_ − x_w_ − a_w_ data can be applied to dry fruit powders, such as mango and its derivatives [71]. These models will be useful in understanding the physical stability of extracts from the raw material of fruit. The higher the CWC and CWA values, the greater the resistance of the samples to physical deterioration (aggregation of amorphous powder and recrystallisation of sugar), which is favored by water sorption [72,73].

### 3.4. Thermal Stability

**Figure 7:**
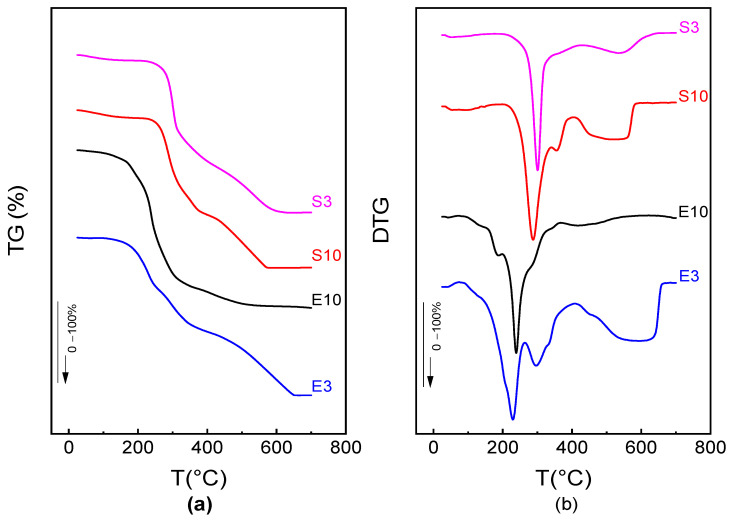
TGA (**a**) and DGTA (**b**) curves for mango peel extracts (E3; E10) and seed extracts (S3; S10) powders.

Figure 7 shows the thermal degradation of samples obtained from mango peel and seed as valorized raw materials, obtained by thermogravimetric analysis (TGA). The TGA curves and their derivatives (DTGA) give information about the mass loss that occurred in the sample as a function of temperature. The degradation of the components in the polymeric matrix is represented by a series of sequential peaks in the thermal degradation pattern shown in the DTGA curves (Figure 7b). Table 3 summarizes the temperature peak of each degradation event and the associated mass loss in the sample. The first mass loss step corresponds to water-bonded vaporization, which appeared at 73 °C for S3 (3.8% mass loss) and S10 (4.5% mass loss) and 86 °C for E3 (2.4% mass loss) and E10 (4.5% mass loss). The samples exhibited a major degradation peak (a doublet in some cases) at approximately 200 and 300 °C, respectively, for E and S extracts, which is in the range reported by Reinoso et al. [74] for compounds of high molecular weight such as xanthan gum. Subsequent degradation steps were also observed at temperatures higher than 400 °C for the different extracts that could be attributed to the degradation of long-chain, high molecular weight polymers [75,76] or to secondary degradation. The higher temperature of the main peak of the seed extract samples suggests the higher thermal stability of their components.

The peel extracts exhibited different peak splitting, suggesting the presence of compounds with different thermal sensitivity. Polysaccharide pyrolysis begins with the random break of glycosidic bonds, followed by further decomposition at 200 to 300 °C [77]. The main broad peak decomposition of the powder extract occurred between 300 and 367 °C, with an approximate mass loss of 62 to 74%. This behavior was similar to that obtained for tara gum in a fish gelatin–glycerol polymeric structure, which is found around 300 °C [75]. Finally, the residue represents the fraction of ashes that contain minerals that cannot be degraded, which varies between 2.4 and 7% of the total sample mass. Furthermore, S3, S10, and E3 are observed to show a degradation curve between 500 and 600 °C, which is mainly attributed to the composition of free fatty acids and volatile compounds. Among the lipids that degrade at these high temperatures are triglycerides, phospholipids, and sterols [76].

## 4. Conclusions

Mango peel and seed can be valorized, minimizing the environmental impact of fruit processing waste by obtaining aqueous extracts of nutritional interest for the food industry. The dried extracts from the skins are rich in carbohydrates, while those from the seeds contain more proteins and lipids. The effect of extraction pH was not significant for the composition and hygroscopic nature of the extract. Dry peel extracts showed a higher water adsorption capacity, with a critical moisture content for the change to a rubbery state at room temperature of about 3%, implying the need for careful handling to prevent powder stickiness. The seed extracts were less sensitive to hydration, in line with the high lipid content of low-melting temperatures (peak at 18 °C) and proteins. Both extracts contained compounds with good thermal stability, with degradation starting above 175 °C. Thermogravimetric analysis offers valuable insights that find practical applications in the industrialization of extracts derived from mango residues. This analytical technique aids in comprehending the composition and stability of these extracts, thereby facilitating enhancements in production processes and ensuring stringent quality control measures. Such measures are pivotal for both the viability and the superior quality of mango-derived industrial products. Therefore, these can be used in the formulation of food products, even in a thermoprocessed form, thus minimizing the environmental impact of fruit residues.

## Figures and Tables

**Table 1:** Sample composition of mango seed extracts (S3 and S10) and peel extracts (E3 and E10).

Sample Code	Moisture %	* Ash %	* Lipids %	* Carbohydrate %	* Proteins %
E3	13.16 ± 0.99 ^a^	9.30 ± 0.61 ^a^	2.93 ± 0.01 ^a^	73.44 ± 1.20 ^b^	0.35 ± 0.03 ^b^
E10	11.24 ± 0.54 ^d^	9.10 ± 0.71 ^a^	3.18 ± 0.31 ^a^	75.95 ± 0.54 ^a^	0.37 ± 0.03 ^b^
S3	12.55 ± 0.23 ^c^	8.47 ± 0.46 ^a^	18.56 ± 0.10 ^b^	28.05 ± 0.30 ^c^	32.07 ± 0.2 ^a^
S10	12.96 ± 0.83 ^b^	9.65 ± 0.53 ^a^	17.32 ± 0.58 ^b^	28.16 ± 0.90 ^c^	31.25 ± 0.4 ^a^

* Results expressed on a dry basis; data are expressed as mean ± standard deviation. Different letters in the same column express statistically significant differences (*p* < 0.05).

**Table 2:** Parameters of the fitted Peleg model and coefficient of determination (R^2^) for the moisture uptake kinetics of different mango peel and seed extracts at different RH values.

Samples	Sales	RH (%)	K_1_ (min(g/g db))^−1^	K_1_ (g/g db)^−1^	R^2^	1/K_1_	1/K_2_
E3	LiCl	11.3	322.13 ± 7.49 ^b^	75.03 ± 1.18 ^c^	0.92	0.003 ^a^	0.013 ^n^
	Mgcl_2_	32.78	114.30 ± 7.12 ^d^	27.39 ± 2.34 ^h^	0.97	0.009 ^i^	0.037 ^k^
	K_2_Co_3_	46.16	46.69 ± 8.59 ^h^	18.22 ± 1.35 ^k^	0.95	0.021 ^f^	0.055 ^h^
	Mg(NO_3_)l_2_	52.89	28.26 ± 6.47 ^i^	14.09 ± 1.10 ^m^	0.91	0.035 ^c^	0.071 ^e^
	NaCl	75.28	5.13 ± 0.60 ^l^	3.37 ± 0.11 ^o^	0.97	0.195 ^k^	0.296 ^a^
E10	LiCl	11.3	328.61 ± 16.24 ^a^	144.68 ± 14.12 ^b^	0.89	0.003 ^a^	0.007 ^o^
	Mgcl_2_	32.78	85.42 ± 5.16 ^e^	34.41 ± 2.41 ^g^	0.94	0.012 ^i^	0.029 ^l^
	K_2_Co_3_	46.16	28.50 ± 2.86 ^i^	14.38 ± 0.57 ^m^	0.95	0.035 ^f^	0.070 ^f^
	Mg(NO_3_)l_2_	52.89	18.59 ± 1.53 ^k^	10.45 ± 0.36 ^m^	0.92	0.054 ^c^	0.096 ^c^
	NaCl	75.28	6.01 ± 0.41 ^l^	3.80 ± 0.10 ^o^	0.95	0.166 ^k^	0.263 ^a^
S3	LiCl	11.3	173.20 ± 0.95 ^c^	70.43 ± 5.09 ^d^	0.92	0.006 ^b^	0.014 ^n^
	Mgcl_2_	32.78	59.93 ± 0.15 ^g^	40.05 ± 1.83 ^e^	0.94	0.017 ^d^	0.025 ^m^
	K_2_Co_3_	46.16	28.93 ± 2.05 ^i^	25.55 ± 0.45 ^i^	0.99	0.035 ^f^	0.039 ^j^
	Mg(NO_3_)l_2_	52.89	26.05 ± 3.18 ^i^	21.85 ± 0.66 ^n^	0.97	0.037 ^g^	0.046 ^i^
	NaCl	75.28	16.41 ± 2.81 ^k^	11.67 ± 0.57 ^n^	0.92	0.061 ^j^	0.086 ^d^
S10	LiCl	11.3	172.11 ± 4.17 ^c^	487.25 ± 3.34 ^a^	0.87	0.006 ^b^	0.002 ^p^
	Mgcl_2_	32.78	66.89 ± 13.87 ^f^	37.67 ± 2.47 ^f^	0.92	0.015 ^h^	0.027 ^m^
	K_2_Co_3_	46.16	28.13 ± 2.60 ^i^	24.89 ± 0.55 ^o^	0.98	0.036 ^ef^	0.040 ^j^
	Mg(NO_3_)l_2_	52.89	21.58 ± 2.05 ^j^	16.92 ± 0.39 ^l^	0.99	0.041 ^e^	0.059 ^g^
	NaCl	75.28	16.35± 0.71 ^k^	10.22 ± 0.18 ^n^	0.98	0.061 ^j^	0.098 ^b^

Data are expressed as mean ± standard deviation. Different letters in the same column express statistically significant differences (*p* < 0.05).

**Table 3:** Degradation temperatures and residues from the TGA measurement for the extract of mango peel (E3; E10) and seed (S3; S10) extract.

Degradation Range	Sample			
	E3	E10	S3	S10
*T* Peak 1 (°C)	228–299 ^c^	200–225 ^b^	301 ^a^	293 ^a^
*T* Peak 2 (°C)	591 ^b^	424 ^c^	613 ^a^	615 ^a^
Initiation of degradation (°C)	175 ± 3 ^d^	201 ± 0 ^c^	225 ± 0 ^b^	269 ± 0 ^a^
End of degradation (°C)	641 ± 16 ^b^	635 ± 4 ^b^	650 ± 0 ^a^	650 ± 19 ^a^
Water mass loss (%)	2.4 ± 0.1 ^c^	4.5 ± 0.1 ^a^	3.8 ± 0.1 ^b^	4.5 ± 0.0 ^a^
Peak 1 Mass loss (%)	81 ± 7 ^b^	87 ± 5 ^a^	86.2 ± 0.4 ^a^	79.9 ± 0.2 ^b^
Peak 2 Mass loss (%)	16.4 ± 0.14 ^b^	9.6 ± 0.62 ^a^	9.4 ± 0.11 ^a^	14.6 ± 0.8 ^b^
Residue (%)	2.4 ± 0.8 ^c^	3.2 ± 1.1 ^bc^	4.5 ± 0.2 ^b^	7.0 ± 0.1 ^a^

Data are expressed as mean ± standard deviation. Different letters in the same row express statistically significant differences (*p* < 0.05).

## Data Availability

The data used to support the findings of this study can be made available by the corresponding author upon request.
